# Developing species-specific welfare scoresheets for the African Turquoise Killifish

**DOI:** 10.1038/s41598-026-58465-3

**Published:** 2026-06-23

**Authors:** Elena De Felice, Antonio Palladino, Aniello Vitale, Ferdinando Flagiello, Maria Raggio, Chiara Attanasio, Paolo de Girolamo, Livia D’Angelo

**Affiliations:** 1 School of Biosciences and Veterinary Medicine, University of Camerino, Camerino, Italy; 2 Department of Clinical Sciences and Translational Medicine, Faculty of Medicine and Surgery, University of Rome Tor Vergata, Rome, Italy; 3 Department of Veterinary Medicine and Animal Production, University of Naples Federico II, Naples, Italy

**Keywords:** Laboratory fish, *Nothobranchius furzeri*, Ageing, Welfare, Scoring system, Biological techniques, Ecology, Ecology, Zoology

## Abstract

The African turquoise killifish *Nothobranchius furzeri* has emerged as a valuable vertebrate model for ageing research. Despite its increasing use in laboratory studies, systematic welfare monitoring approaches in *N. furzeri* is still emerging, and only a limited number of structured health-assessment frameworks have been proposed. Here, we present a welfare scoring system designed for daily monitoring of *N. furzeri* across its entire lifespan under laboratory conditions. Building on established zebrafish welfare frameworks and adapting them to the biological and ethological characteristics of *N. furzeri*, we identified a set of morphometric, behavioural and clinical parameters, including body condition score, external appearance, food intake, respiratory pattern, swimming activity, tank occupancy and clinical signs. Each parameter was assigned a numerical score ranging from 0 to 3, reflecting increasing degrees of welfare impairment. We further defined standardized intervention criteria, including humane endpoints, to support decision-making in cases of compromised welfare. Importantly, we established five distinct age stages to enable age-specific welfare assessment in two widely used wild-type strains, GRZ/GRZ-AD and MZM04-10. This scoring system provides a practical, reproducible, and easily implementable tool that can be seamlessly integrated into routine husbandry procedures. Its application is expected to promote standardized welfare assessment, improve experimental reproducibility, and enhance animal welfare standards in research using *N. furzeri*.

**Supplementary Information:**

The online version contains supplementary material available at https://doi.org/10.1038/s41598-026-58465-3.

## Introduction

The increasing use of *Nothobranchius furzeri*, also known as African turquoise killifish, in the pre-clinical setting worldwide calls for the need to define and pursue adequate welfare approaches. Several publications detailing housing and husbandry as well as breeding conditions have been published since 2016^1–7^. These works provided detailed descriptions of routine colony management and experimental husbandry conditions, offering practical guidance for maintaining *N. furzeri* in laboratory facilities and therefore represent a useful framework for developing welfare monitoring approaches. Very recently, Naumann and colleagues^8^ proposed the first killifish health scoring system and routine colony health surveillance program, a valuable tool to comply with ethical and legal requirements when using this fish species. This latter report provides new information on clinical signs and conditions that may occur in young and aged *N. furzeri*, by relying on a “traffic light” system, where green state refers to physiologic conditions with no apparent signs of pain, harm, or distress; yellow state for fish exhibiting clinical signs that indicated short-term mild pain, harm, or distress, and red state for fish showing conditions with acute clinical signs or severe conditions that, being irreversible or progressive, are defined as humane endpoints, thus requiring immediate euthanasia^8^. Despite this undoubtedly valuable and easy-to-use system, the proposed clinical evaluation does not consider age-related clinical signs except for certain health categories/subcategories, i.e. reduced feeding behavior, medium reduced social behavior, and underconditioned body condition^5^.

Compared to mammals^9^, short-lived model organisms are characterized by accelerated life trajectories, causing rapid transitions between developmental and ageing stages. This feature is particularly pronounced in *N. furzeri*, the shortest-lived vertebrate animal model^10^. Consequently, age specific welfare evaluation is especially important in this species since physiological and behavioral baselines can change dramatically over brief periods of time. Studies employing *N. furzeri* as a research model are often targeted to study longevity or lifespan, thus focusing on healthy ageing or interventions to extend lifetime^11^. This experimental approach requires that subjects are followed over the entire life until either spontaneous death occurs or euthanasia is deemed necessary due to the severity of clinical signs. Therefore, welfare assessment must include parameters such as activity, body condition, feeding behaviour, and general physiological status, which can vary drastically from age to age. For instance, juveniles at sexual maturity possess baseline values differing from those of old or geriatric subjects, as also demonstrated at the molecular level, where Mazzetto and coworkers reported a sharp gene differential expression over these time-points^12^.

We strive to identify corresponding time points in *N. furzeri*, based on the phenotypic hallmarks of the age stages coupled with molecular traits, to develop a punctual clinical and welfare assessment. We focused on two of the most used captive strains, GRZ/GRZ-AD and MZM-0410, the short- and long-lived, respectively. It is of note that fish belonging to the two strains show a near twofold difference in lifespan, but similar growth and maturation rates^12^. The approach herein reported can be easily translated to the other short- or long-lived strains of this teleost species (i.e. ZMZ1001, MZCS, etc.).

Morphological, behavioral, and clinical characteristics of *N. furzeri* individuals vary significantly during the course of their lives as a result of physiological aging, which presents a significant obstacle to welfare evaluation. This is even more remarkable for studies on longevity and healthy aging, where animals are tracked over time, and age-related changes need to be separated from signs of poor health and welfare. Therefore, an age-related approach is necessary for the creation of a species-specific welfare evaluation instrument for *N. furzeri.*

The main goal of this report is to advance welfare assessment in *N. furzeri* by developing a practical, species-specific tool for daily monitoring in laboratory facilities. To this end, we drew on existing literature on other laboratory fish, including zebrafish body condition scoring (BCS) systems and welfare scoresheets^13,14^, as well as on the available scientific literature on *N. furzeri*, largely focused on behavioural studies. This approach allowed us to identify piscine welfare indicators that can be adapted to this species while accounting for its specific biological and ethological traits. For example, we excluded group behaviour indicators such as shoaling or schooling, which are commonly used in zebrafish welfare assessment but are not appropriate for this species. From an ethological perspective, *N. furzeri* displays pronounced territorial and courtship behaviours, with males displaying conspicuous coloration and enlarged fins, traits likely shaped by sexual selection and involved in social interactions during reproduction^15^.

Rearing density has been shown to influence total body length, locomotor activity, boldness, aggressiveness, and feeding behaviour in *Nothobranchius furzeri*^16^. Consideration of these housing conditions is therefore essential for accurate welfare assessment.

## Methods

### Housing and husbandry

The animal study protocol was approved by the Italian Ministry of Health protocols n° 77/2023-PR and 1138/2024-PR. All procedures adhered to relevant guidelines and regulations, conforming to the ARRIVE guidelines^17^. All fish were maintained in the ZENOLab fish facility of the University of Naples Federico II (Italy). The establishment was authorized by the Italian Ministry of Health (Competent Authority) according to D.vo 26/2014. Animals were housed in a recirculating water system (RS) (Tecniplast, Buguggiate, Italy). Tank allocation in our facility was organised as follows: up to 5 females were housed per 8-L tank, up to 3 females together with 1 male were housed per 8-L tank, and males were also housed singly in 3.5-L tanks. Concerning microenvironment, optimal water parameters were: temperature between 26 and 28 °C; neutral pH (7–7.5); conductivity value of approximately 660 µS; water hardness (as CaCO_3_) equivalent to 400 mg/L; ammonia (NH3/NH4) 0.01–0.1 ppm; nitrite (NH_2_-) < 0.2 ppm; nitrate (NO_3_-) < 50 ppm. The remote control of photoperiod was set at 12 light/12 dark hours, with a gradual decrease and increase in light intensity over approximately 15–30 min mimicking dusk and sunrise with the artificial white light (range of lux: 50–250 lx).

After hatching, newly hatched juveniles were immediately fed with live *Artemia nauplii*. The daily ration was approximately 2 mL per animal, split into three feedings per day, until weaning at 21 days post-hatch. Fish were fed frozen *Chironomus* spp. or commercial dry feed GEMMA Micro - SKRETTING (Nutreco company), depending on routine husbandry practices within the facility. Juveniles were fed a mixed diet of *Artemia nauplii* and frozen *Chironomus spp.* or *Artemia nauplii* and dry feed (pellet size 0.3–0.4 mm); by 8 weeks of age, they were maintained on *Chironomus* or dry feed alone (pellet size 0.7–0.8 mm). The type of feed was not considered an experimental variable, as the aim of the study was to develop a welfare assessment framework rather than to evaluate nutritional effects. For juveniles and adults, feeding was adjusted based on appetite and feeding behaviour, ensuring that food was consumed within 5 min after administration. The approximate daily ration corresponded to ~ 5% of body weight under our facility conditions.

Animals subjected to procedures such as body weight measurement were anaesthetized with buffered tricaine methanesulfonate (MS-222), 100 mg/L. For animal killing, an overdose of lidocaine hydrochloride: 1500 mg dissolved in one liter of water system (between 26 and 28 °C) buffered with 2 g/liter NaHCO3 per single animal, and the exposure to the solution was performed at room temperature.

### Determination of the number of opercular movements

Respiratory rate (opercular beat) was measured for each fish over a 1 min period in its home tank to avoid handling- or novel environment-related stress, as well as measuring the baseline under controlled environmental conditions. Three individuals per age stage were included in the analysis. Video recordings were obtained using a digital camera (Logitech C720) positioned in front of the tank to minimize disturbance.

For each individual, a 60s video of opercular movements was recorded and subsequently analyzed using video-editing software (Movie Maker - Windows). Playback speed was reduced to facilitate accurate counting, and the number of opercular movements per minute was determined.

Counts were performed independently by three observers.

### Inspections and scoring of abnormal phenotypes

For welfare assessment of adult fish, we included parameters listed by Naumann and colleagues^8^ for *N. furzeri* and implemented and adapted those commonly reported in other fish species^14^, including zebrafish^18,19^. Identified parameters comprise Body Condition Score (BCS), appearance, food consumption, respiratory pattern, swimming activity, tank occupancy, and clinical signs. Based on these parameters, we developed a scoresheet for general health assessment (Tables 1 and 2). For each parameter, a score is given for each point of age as follows: score = 0 none (physiologic conditions); score = 1 mild; score = 2 moderate; score = 3 severe. The severity assessment must be made for single and cumulative scores (Table 3).

**Table 1:** Morphological parameters scoresheet for general health assessment.

Parameters	EARLY JUVENILE 1dph – 21dph	Young adult 3/4wph – 8/9wph	Adult 9/10wph – 24/25wph	Old 25/26wph – 38/39wph	Very Old over 39/40wph
Body Condition Score					
Emaciation, loss of weight < 20%	3	3	3	2	1
Under-conditioning	3	3	2	1	1
Well-conditioned	0	0	0	0	0
Over-conditioned	0	1	1	1	1
Obesity, increase of weight > 20%	N/A	3	3	2	N/A
Appearance					
Decolouration of the skin or scale patterning	N/A	3	3	1	0
Caudal fin altered pigmentation/integrity	3	3	2	1	0
Operculum (missing/lesions/etc) - Gill hyperemia	N/A	3	2	2	1
Missing or raised scales	0	3	3	3	3
Eyes (exophtalmos/lens opacity/etc.)	3	3	3	1	0
Skeletal deformations (i.e. mandibular deformities, spinal curvature)	3	3	3	1	1
Partial or intermittent loss of balance (Vertical swim)	2	2	2	2	3
Circling temporary	2	2	1	1	1
Surface swim/deep swim	2	2	1	1	0
Total loss of balance	3	3	3	3	3

**Table 2:** Physiological parameters scoresheet for general health assessment.

Parameters	EARLY JUVENILE 1dph – 21dph	Young adult 3/4wph – 8/9wph	Adult 9/10wph – 24/25wph	Old 25/26wph – 38/39wph	Very Old over 39/40wph
Food consumption					
Normal	0	0	0	0	0
Unresponsive to the usual food (1 day)	1	1	1	1	0
Unresponsive to the usual food (up to 4 days)	3	3	3	2	1
Unresponsive to the usual food (˃ 4 days)	3	3	3	2	1
Respiratory pattern					
Increased opercular beat-rate	N/A	2	2	3	3
Decreased opercular beat-rate	N/A	2	2	2	1
Swimming activity					
Increased swimming speed	1	1	1	N/A	N/A
Decreased swimming speed	3	3	2	0	0
Lethargic, no reaction to external stimuli	3	3	3	1	0
Partial or intermittent loss of balance (Vertical swim)	2	2	2	2	3
Circling temporary	2	2	1	1	1
Surface swim/deep swim	2	2	1	1	0
Total loss of balance	3	3	3	3	3
Tank occupancy					
Swimming through the water column	0	0	0	0	0
Swimming in the 1/3 of the rear of the tank	2	2	2	1	0
Immobility in the rear angles of the tank	3	3	2	1	1
Systematic swimming on the tank bottom	1	1	1	1	0
Clinical signs					
Poor growth	3	2	2	N/A	N/A
Cutaneous mucus hypersecretion	N/A	2	2	1	1
Petechiae and/or hemorrhage	3	3	3	3	3
Distended abdomen	N/A	2	2	1	1
Skin lesions (ulcers, neoformations, hyperplasia)	3	3	3	3	3

**Table 3:** Scoring of animal welfare in consideration of animal age.

**Early juvenile - Young Adult - Adult**
**0–3** Normal.
**4–8** Monitor carefully. Consider veterinary treatment including analgesics and consider also to analyse water quality.
**9–12** Suffering, provide relief, consult the specialized veterinarian, and consider euthanasia.
**13–18** Severe status, euthanasia, rethink experimental procedure or management.
Euthanasia may be considered if a score of 3 is observed in any of the categories, except for behaviour-related categories when considered in isolation.
**Old - Very Old**
**0–3** Monitor carefully.
4-8 Suffering and provide relief, consult the specialized veterinarian, and consider euthanasia.
**> 8** Severe status, euthanasia, rethink experimental procedure or management.

### Body Condition Index and Specific Growth Rate

**Fig. 1:**
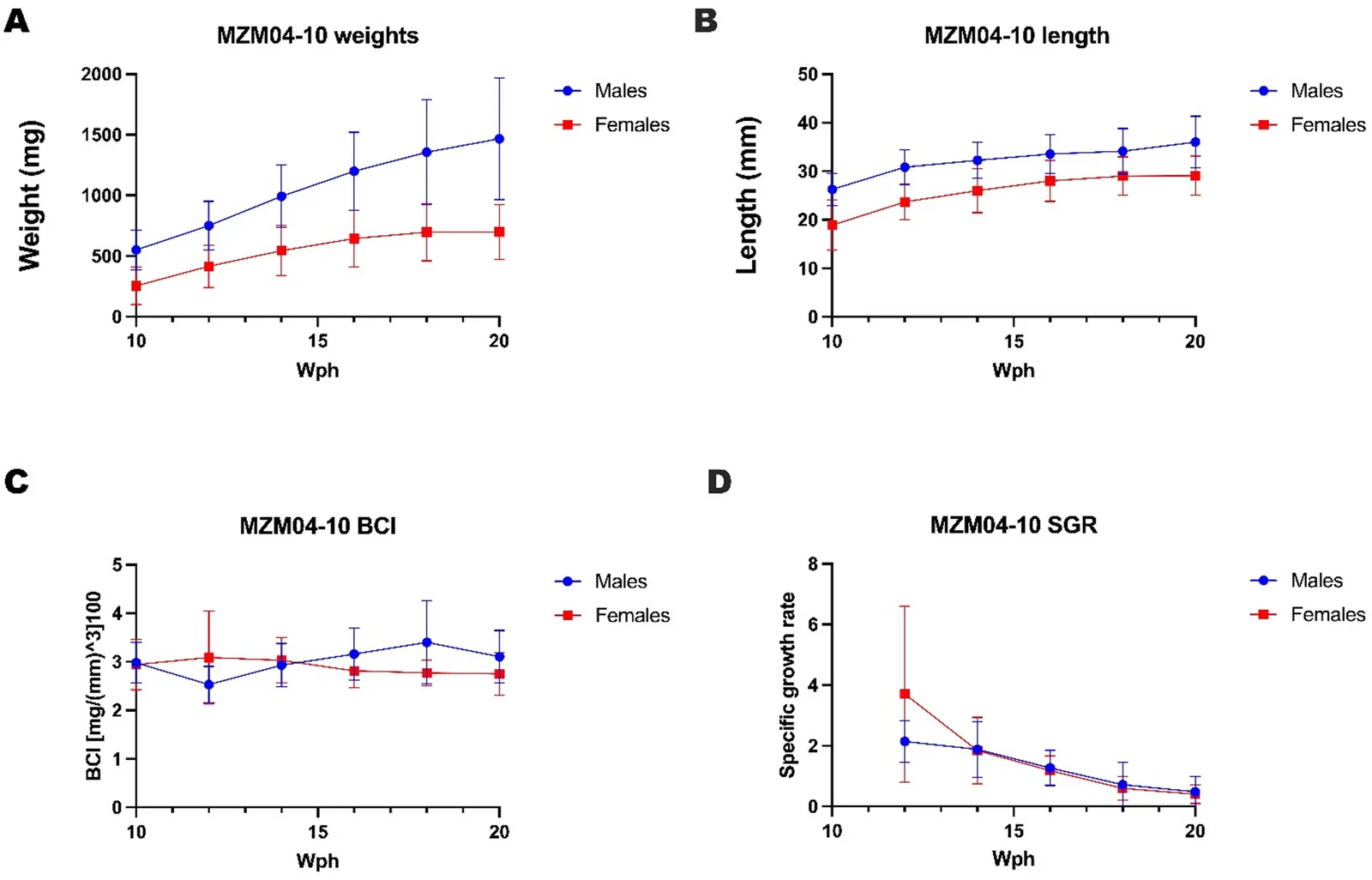
Morphometric measurements, body condition index (BCI), and specific growth rate (SGR) of the MZM strain. (**A**) Longitudinal bi-weekly measurements of two cohorts (20 animals in total) of male and female specimens belonging to the MZM-0410 strain were conducted from week 10, when breeding begins, until week 20 of age. Males weighed about 1.5 g, while females weighed approximately 600 milligrams. (**B**) Males reached an average total length (from the anterior tip of the head to the posterior end of the tail) of up to 35 mm, whereas females reached an average total length of up to 28 mm. **C.** The body condition index (BCI), used to assess the overall health status of males and females, was calculated as the ratio of weight (mg) to length (mm)³. **D.** The specific growth rate (SGR) indicates the growth pattern, which tends to decrease over time due to the slowing of growth in adulthood.

**Fig. 2:**
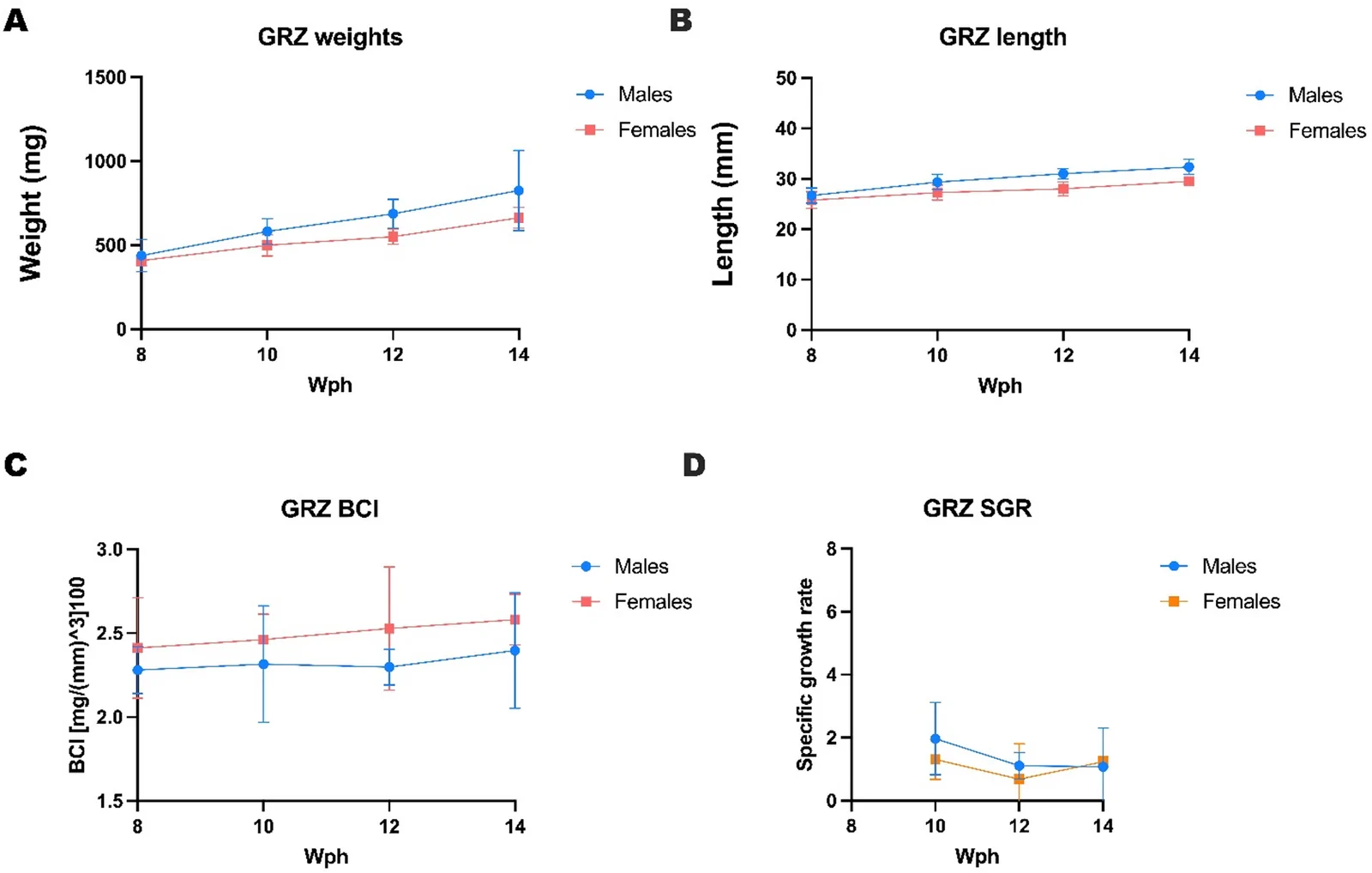
Morphometric measurements, body condition index (BCI), and specific growth rate (SGR) of GRZ strain. **(A)** Longitudinal bi-weekly measurements of two cohorts (20 animals in total) of male and female specimens belonging to the GRZ-AD strain were conducted from week 8, when breeding begins, until week 14 of age. Males weighed just under 800 milligrams, while females weighed around 600 milligrams. **(B)** Males reached a total length (from the anterior tip of the head to the posterior end of the tail) of up to 30 mm on average, whereas females reached up to 28 mm on average. **(C)** The body condition index (BCI), used to assess the overall health of males and females, was calculated as the ratio of weight (mg) to length (mm)³. **(D)** The specific growth rate (SGR) differed between males and females, tending to decrease in males, while in females it decreased initially but increased again between 12 and 14 weeks of age.

The calculation of Body Condition Index (BCI) and Specific Growth Rate (SGR) enabled us to establish specific reference values that provide a practical baseline and identify objective parameters for welfare assessment in *N. furzeri*. Figures 1 and 2 show weekly BCI and SGR values for the MZM-0410 and GRZ/GRZ-AD strains, respectively. Growth and body size values observed in our study follow patterns reported in the literature for *N. furzeri*, although absolute values may vary across facilities due to differences in environmental conditions and husbandry practices^20,21^.

It is important to note that both indices are influenced by multiple factors, including: age (younger fish typically exhibit higher growth rates and energy demands); diet (quality, quantity and feeding frequency); environmental conditions such as temperature (extremes alter metabolic rates and energy storage), light (which can affect feeding behaviour) and water quality (e.g., dissolved oxygen and pH); stocking density (overcrowding increases competition and stress); infections or disease^22^. Consequently, routine monitoring and control of these factors are essential for effective colony management.

Another morphometric index is the Body Condition Score (BCS) - a simple, rapid, and non-invasive tool used to assess body condition as an indicator of general health^23^ across species. In laboratory fish, body condition scoring systems have been most extensively developed for zebrafish^24^.

During the experimental projects, we had the opportunity to record the morphometric measurements of GRZ/GRZ-AD and MZM-0410 strains over their lifespan, which were used to calculate BCI and SGR for the two strains, thus setting a reference. These parameters have turned fundamental also to develop the scoring system to evaluate the body condition.

Morphometric measurements were obtained every two weeks under anaesthesia. In the MZM04-10 strain, measurements were collected from 10 to 20 weeks of age, while in the GRZ/GRZ-AD strain they were collected from 8 to 14 weeks of age. At each time point, fish were gently collected with a net and weighed individually on a precision balance using a Petri dish. Total length was measured from the tip of the rostrum to the distal end of the caudal fin by placing the anaesthetized fish on millimeter paper and measuring with a ruler.

The BCI, often represented as Fulton’s condition factor (K), is a measure reflecting energy reserves and general body condition in an organism. In addition, the index normalizes growth across individuals of different sizes, facilitating direct comparisons between experimental and control groups. It is typically calculated using the relationship between weight and length, by applying the following formula^25^:

$$\:BCI:\:\frac{Wet\:Body\:Weight\:\left(mg\right)}{Total\:body\:length\:\left(m{m}^{3}\right)}\times\:\:100$$

The SGR is a measure used to evaluate the growth performance of fish over a specific period. It is expressed as a percentage of weight gain per day and is calculated using the formula:

$$\:{SGR}_{j-i}\:\frac{{ln}{X}_{j}-{ln}{X}_{i}}{{I}_{j}-\:{I}_{i}}$$

where *X*_*i*_ and *X*_*j*_ represent the mean wet body weight for each diet at the beginning and end of the period, respectively, and t_i_ and t_j_ represent the time in days of the beginning and end of the period, respectively^26^.

### Behavioural setup

To assess swimming activity in this species, we analyzed 10 subjects (male: female ratio 1:1), representing young, adult and unhealthy individuals. For each trial, the home tank was moved out from the recirculating housing system to a designated recording area. This approach was used to avoid handling-related stress to the animals as well as minimize animal reflections on the tank wall during recording. Additionally, in order to minimize the stressor effect due to displacement, a 60-minute acclimatization period was provided for each animal^27^. Subsequently, the animals were observed and recorded individually for 5 min. Five minutes are an interval sufficient to detect representative behaviors and, at the same time, ensure operational sustainability of the workflow within the facility, likely representing the time a facility operator allocate to the animal observation required for the daily monitoring activities. We quantified swimming activity and tank occupancy by measuring time spent mobile vs. immobile across the water column, mean swimming speed and total distance travelled. To capture these metrics, we used two camera views (frontal and overhead), allowing a more accurate assessment of both horizontal movement and vertical tank occupancy, with Logitech C270 cameras (720p/30 fps). Data were recorded, tracked and analyzed using the ANY-maze behavioral analysis software^41^, version 7.49 (64-bit; Stoelting Co., Wood Dale, IL, USA).

### Applicability

#### Level 1 — Direct use

daily checks by animal keepers, animal-care technologists and veterinarians for standard and post-procedural monitoring.

#### Level 2 — Forward-looking

a basis for discussion at the science–regulation interface.

## Results

### Definition of time-points for an age-related welfare assessment

We first defined the time points for our welfare-scoring system. To capture age-related changes across *N. furzeri* lifespan, we reviewed the literature on short- and long-lived strains and selected time points accordingly.

For the short-lived GRZ/GRZ-AD strain, studies commonly refer to the age of 6, 12, 18 and 24 weeks post-hatching (wph)^28–30^. For the longer-lived MZM-0410 strain, typical time points considered are 6, 12, 20, 27, and 39 wph^12^.

For the welfare assessment, we also included stages earlier than sexual maturity, as shown in Table 4:

**Table 4:** Commonly used time points of short- and long-lived strains of *N. furzeri*.

Strain	Age-points				
	Early juvenile stage	Young adult	Adult	Old	Very old
GRZ/GRZ-AD	1–21 dph	3/4–7/8wph	8/9–12/14wph	14/15–20/22wph	over 22wph
MZM04-10	1–21 dph	3/4–8/9wph	9/10–24/25wph	25/26–38/39wph	over 39/40wph

dph: day post hatching; wph: week post hatching.

### Body parameters

**Fig. 3:**
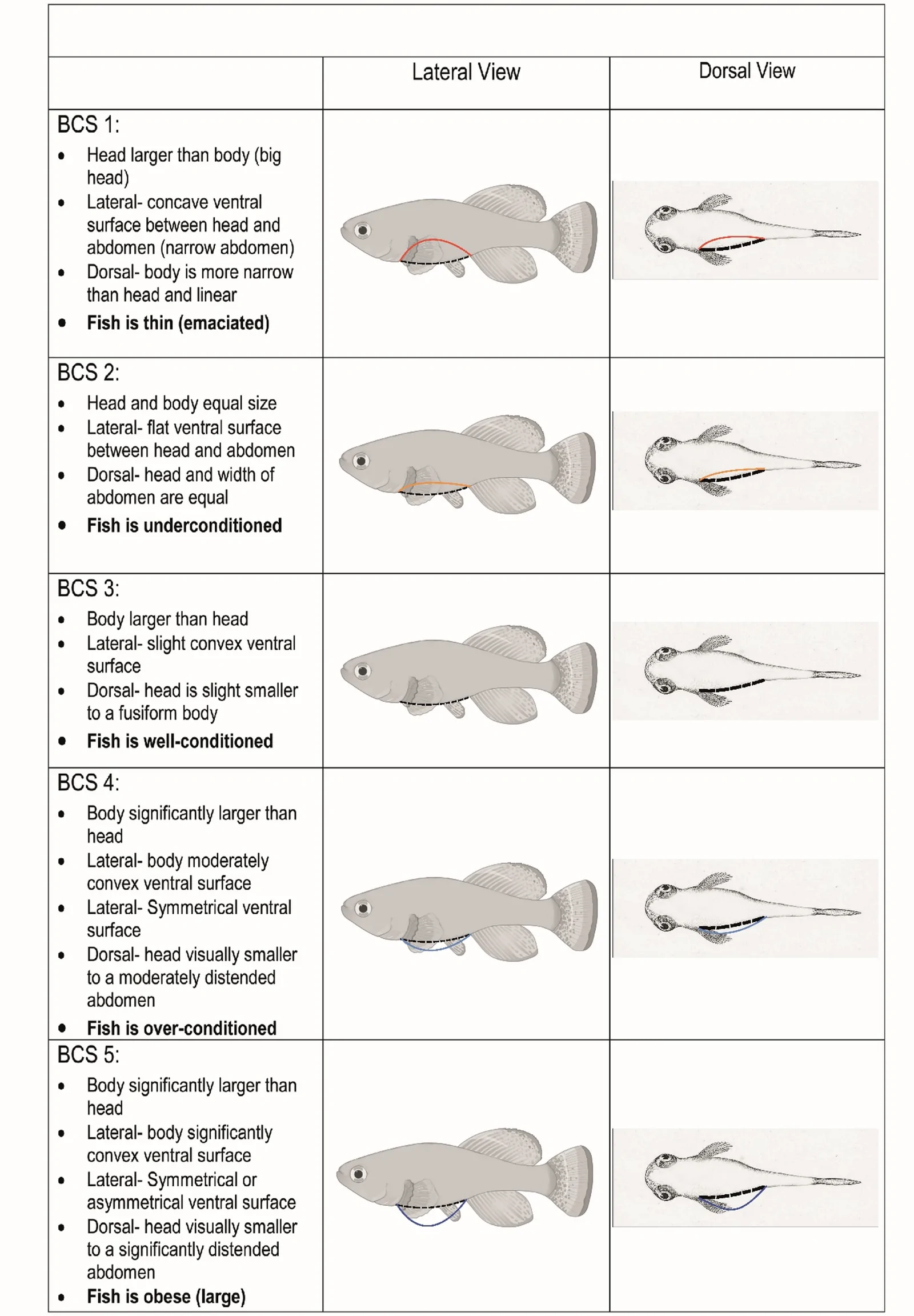
Body Condition Score for the African turquoise killifish. Lateral and dorsal views of adult specimens showing the five body condition scores (BCS), from 1 (emaciated) to 5 (obese), with 3 indicating good health; the dashed line marks the ventral physiological margin of the abdomen, orange lines indicate abdominal pinching (BCS 1–2), and blue lines indicate abdominal bloating.

Body Condition Index (BCI) and Specific Growth Rate (SGR) are the two parameters we have used to assess *N. furzeri* health and growth rate and are adapted to the diagram-based chart proposed for zebrafish^24^ with regards to the morphological characteristics of *N. furzeri* (Fig. 3). According to this scheme, fish are classified into five categories based on abdominal and head appearance and the abdomen-to-head size ratio. Anatomical landmarks for the abdomen vary slightly by view: in lateral view, the region of interest is approximately midway between the pectoral and anal fins; in dorsal view, it is approximately midway between the pectoral and dorsal fins.

Body Condition Score for the African turquoise killifish:

BCS 1 — Emaciated. Fish show a pinched abdomen and marked thinning of the back and nape. This condition warrants close monitoring during development and should, in any case, be considered a humane endpoint.

BCS 2 — Under-conditioned. The hallmark is a pinched abdomen. With regular feeding and a gradual increase in ration, fish may recover to BCS 3.

BCS 3 — Well-conditioned. Fish exhibits a fusiform body with a gently convex abdomen.

BCS 4 — Over-conditioned. Characterized by a distended (“pot-bellied”) abdomen. This may reflect a recent large meal or, alternatively, ascites associated with renal or hepatic disease, which becomes relatively common with age^10,31^. Sexually mature females carrying eggs may show abdominal swelling, most evident near the vent.

BCS 5 — Obese. Fish displays a markedly distended abdomen with increased fullness of the nape and back. This condition is primarily observed in adults and is uncommon in old or geriatric individuals. This condition may reflect excessive energy intake; however, similarly to BCS 4, it may also be associated with pathological conditions (e.g., fluid accumulation or organ dysfunction).

**Fig. 4:**
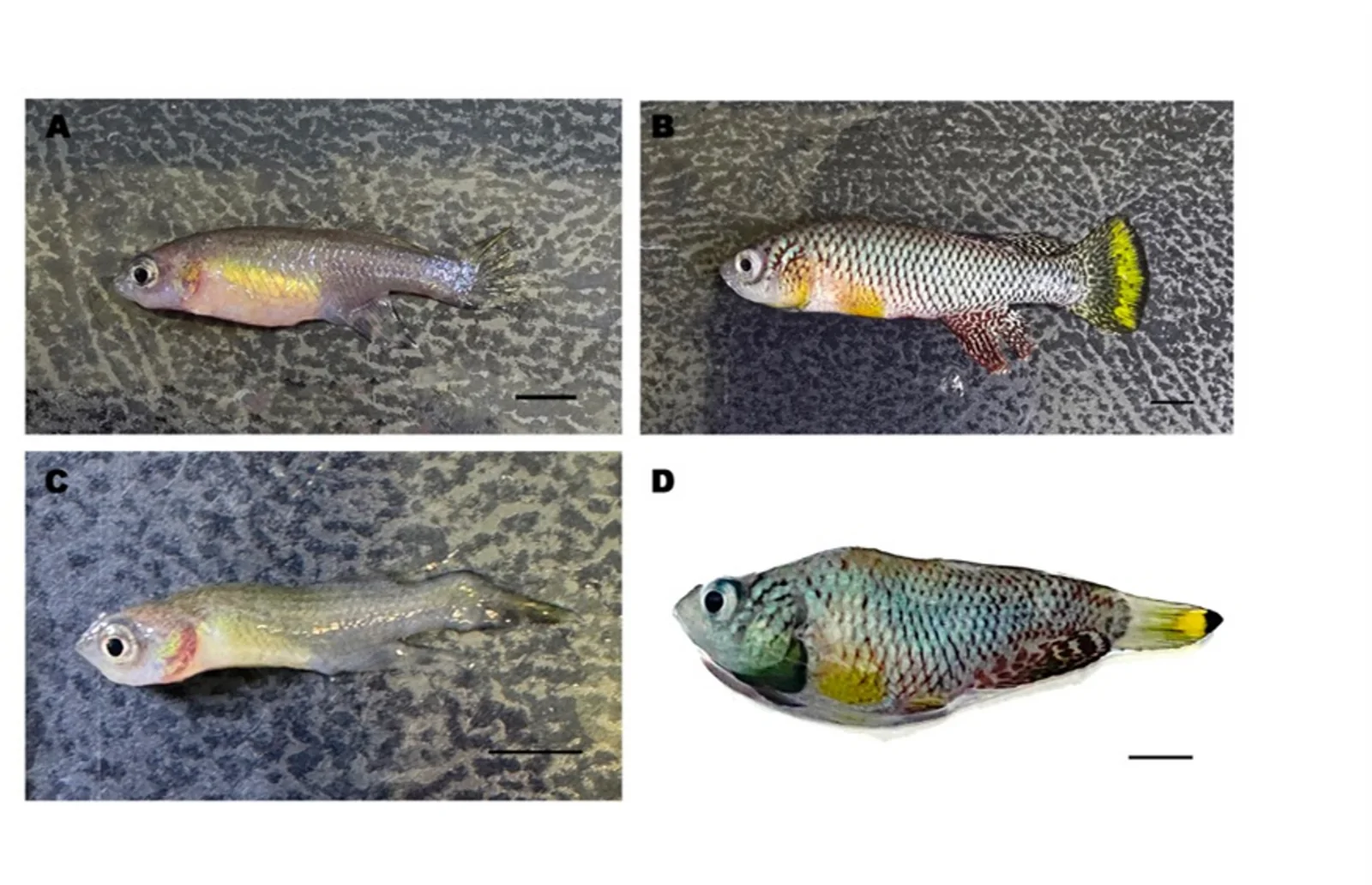
Example of Body Condition Score for the African turquoise killifish. (**A**) Lateral view of an adult female with BCS 3. (**B**) Lateral view of an adult male with BCS 3. (**C**) Lateral view of an adult female with BCS 1. (**D**) Lateral view of an adult male with BCS 5. *Scale bars*: 500 μm.

This BCS provides a more objective rubric than qualitative judgments (e.g., “too thin,” “obese”) and, importantly, relies on a non-invasive assessment: fish can be evaluated from a distance in their home tank without disturbance. Figure 4A-D show examples of different BCS.

### Appearance

**Fig. 5:**
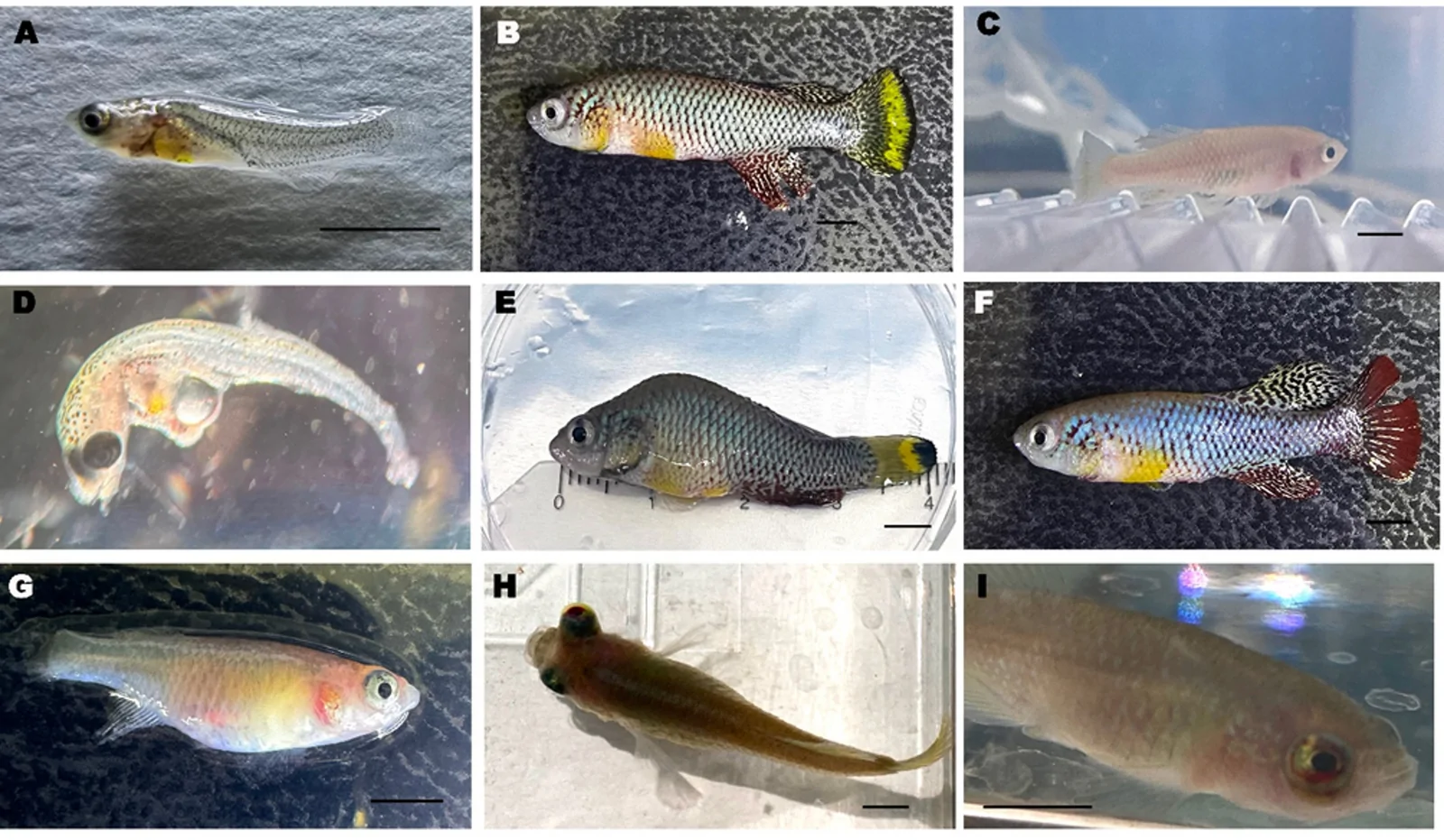
Appearance of African turquoise killifish. **(A)** Early juvenile showing its transparent body with only a few dark spots. **(B)** A young male exhibiting a brilliant colouring of the skin. **(C)** An elderly female displaying the typical loss of colour due to old age. **(D)** Larva at 1 day post hatching with column deformity. **(E)** Adult male affected by spinal curvature. **(F)** Adult male showing loss of rays in the caudal fin. **(G)** Adult female with operculum lesion. **(H)** Adult female diagnosed with right exophthalmos. **(I)** Adult female presenting iris haemorrhage in the right eye. *Scale bars*: 500 μm; D: 200 μm.

Direct observation is the primary indicator for assessing health status. At the early juvenile stage, fish are transparent with only a few dark spots (Fig. 5A), whereas skin coloration develops later, from sexual maturity onward (5–6 wph; Fig. 5B). In old and very old stages, coloration is physiologically altered^32^ and should not be interpreted as a sign of impaired welfare (Fig. 5C). The caudal fin is a useful indicator of age-related changes in pigment metabolism: in some older individuals altered coloration and discrete pigment spots (e.g., reddish or blackish) may be observed.

Body deformities in early juvenile or young stages (Fig. 5D) negatively affect welfare and fitness and should be scored 2 or 3; these animals require close follow-up. By contrast, spinal curvature or general body deformities (Fig. 5E) are typical of old and very old fish and may be scored 1—or even 0 in very old individuals.

Additional parameters to consider include the integrity of the caudal fin (Fig. 5F) and of the operculum (Fig. 5G). The eyes are also informative: lens opacity, consistent with cataract, is frequent in old specimens, whereas exophthalmos (Fig. 5H) or hyphaema (Fig. 5I) can markedly compromise welfare and therefore require close monitoring regardless of age stage.

### Food consumption

Food consumption was assessed during routine feeding sessions under undisturbed home-tank conditions. In line with established *N. furzeri* husbandry practices, feeding response was evaluated within a 5-minute observation window following food administration^2^. Appetite and food intake are key indicators of welfare and must be interpreted in relation to age stage. Healthy juvenile and adult *N. furzeri* are typically voracious, whereas food intake tends to decrease with age. Older and geriatric individuals may display reduced feeding motivation and slower feeding responses compared with younger fish.

### Respiratory pattern

Opercular beat rate is a key clinical and physiological indicator of health and welfare; both decreases and increases should be considered adverse clinical signs requiring attention. We provide reference ranges of opercular beats per minute under controlled laboratory conditions (≈ 26–28 °C, normoxia, unstressed fish) across different age stages. The calculated range in young/adult *N. furzeri* is 100–190 beats per minute (bpm), in adults 90–120 and in old individuals 80–150.

At the early juvenile stage, opercular movements are present but may be difficult to quantify reliably due to small body size and increased locomotor activity; therefore, this parameter should be interpreted with caution in this age group.

A reduced opercular beat rate, particularly in old and geriatric individuals, may reflect age-related physiological decline but should still be evaluated in the context of other clinical signs. Conversely, an increased opercular beat rate in young and adult fish should be considered a potential early indicator of distress, possibly associated with environmental, experimental, or pathological factors.

In Supplementary Video 1, gasping, characterized by short, laboured breaths, often at the water surface, is shown. This behaviour represents a severe adverse clinical sign and a potential humane endpoint, indicative of respiratory distress. It may result from impaired water quality (e.g., hypoxia, elevated ammonia or nitrite), inappropriate temperature, or underlying disease, and should trigger immediate welfare assessment and corrective action.

### Swimming activity

A clear pattern of age-dependent changes in mobility and swimming speed is observable and consistent with data reported in literature^33^. Mobility is defined as movement, even if the center point of the animal is not moving, i.e., rotation of the fish or small fin movements. Young and adult fish show higher locomotor activity, characterized by greater total distance travelled (approximately 4000 cm in 30 min), higher speed velocity (approximately 2 cm/s in 30 min), and more frequent transitions between tank zones^33^. Old and very old animals are pretty much immobile, which is defined as moving less than 0.1 cm/s for at least 1 s^33^. Furthermore, the housing conditions, such as animal densities and environmental enrichment (EE), may influence the swimming activity of young and adult fish. For instance, solitary female fish display a higher swimming velocity compared to fish from high densities^16^.

**Fig. 6:**
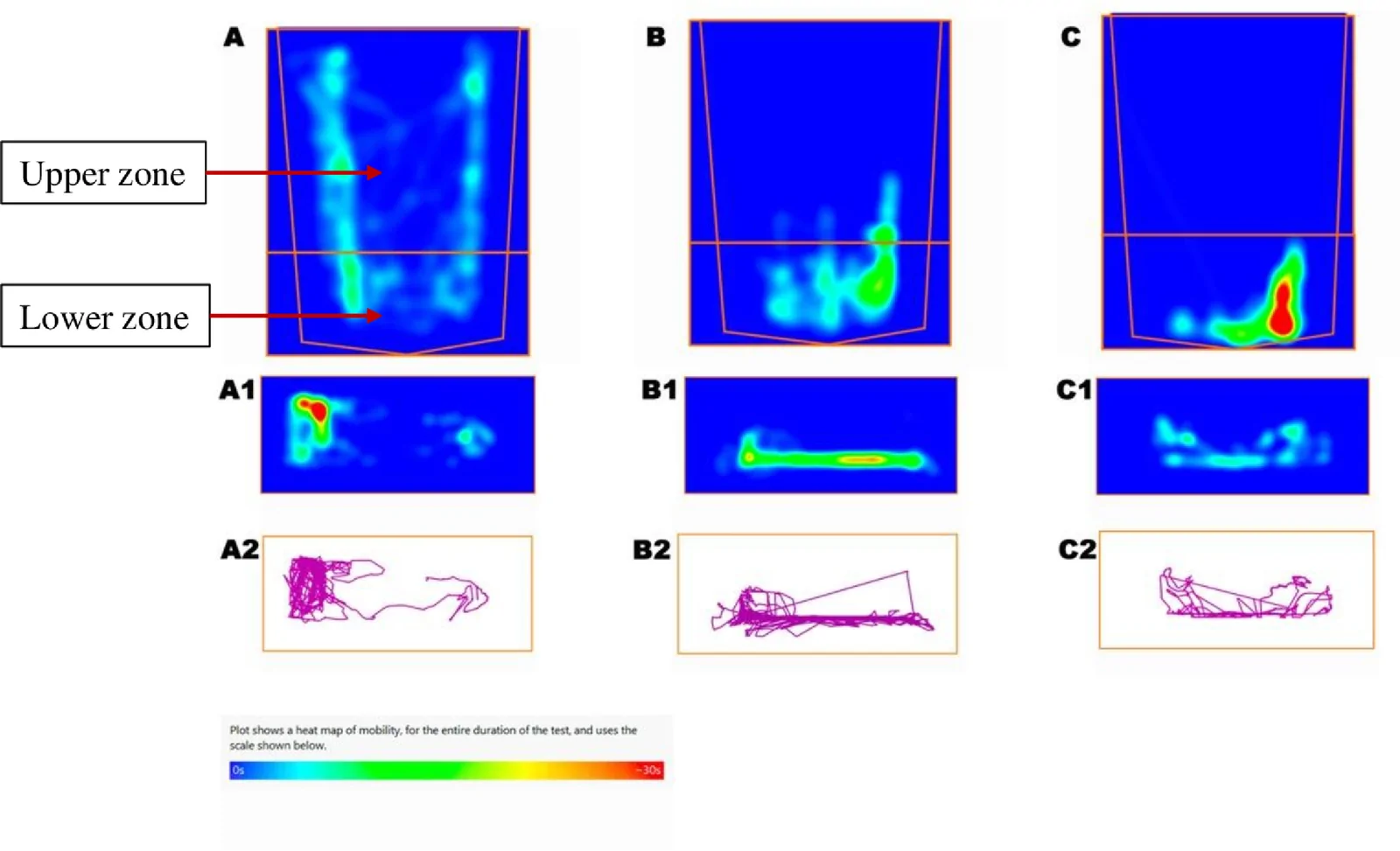
Heat maps of tank occupancy and swimming behaviour over the entire test period (5 min). **A**,** B**, and **C** depict a young, adult and unhealthy individual in front of the tank, respectively. Arrows indicate the upper and lower zones the tank is conceptually divided to track the fish tank occupancy. **A1**,** B1**, and **C1** show the same result but with an up-located camera. These images show a gradual decrease in the distance traveled over the entire water column as fish get older or have poorer health. Graphs **A2**,** B2**, and **C2** illustrate the positions during the test of young, adult, and unhealthy individuals, respectively, display the positions of young, adult, and unhealthy individuals during the test, demonstrating a progressive reduction in the perimeter traversed from young to adult to unhealthy fish.

Geriatric (Supplementary Video 2) or unhealthy individuals may show a lethargic-like state (Fig. 6C2) compared to young individuals (Supplementary video 3). In geriatric fish, however, persistent loss of balance is common and should be treated as a humane endpoint. Altered buoyancy can be identified by abnormal body orientation - often a diagonal posture with the head tilted toward the surface - suggesting impaired buoyancy control (Supplementary Video 4).

### Tank occupancy

In *N. furzeri*, homebase behavior can be described as the establishment of a preferred spatial zone - typically located along boundaries or in sheltered regions - that serves as an anchoring point for movement^33^. From this location, young/adult fish perform repeated short excursions into more central areas and then return to the same site, generating a cyclic exploration–return pattern (Fig. 6A–A2). This behaviour is less evident in adult fish (Fig. 6B-B2). With aging, the spatial organization becomes less defined: older fish tend to spend more time in the center zone at the bottom of the tank (Fig. 6C-C2, Supplementary Video 5). Healthy fish spend most of the time in the front and/or in the center of the tank. Young animals (Fig. 6A) tend to occupy the entire water column while adults (Fig. 6B) mainly stay in the lower half of the tank. Unhealthy fish are generally placed on the rear of the tank or even in one of the rear tank angles (Fig. 6C, Supplementary Video 6). Tank occupancy may represent a useful behavioural indicator of health and welfare, as spatial distribution and use of space can reflect the animal’s response to environmental and husbandry conditions^34,35^. Generally, old or geriatric subjects tend to occupy the bottom of the water column in the rear of the tank (Supplementary Video 2).

### Clinical signs

**Fig. 7:**
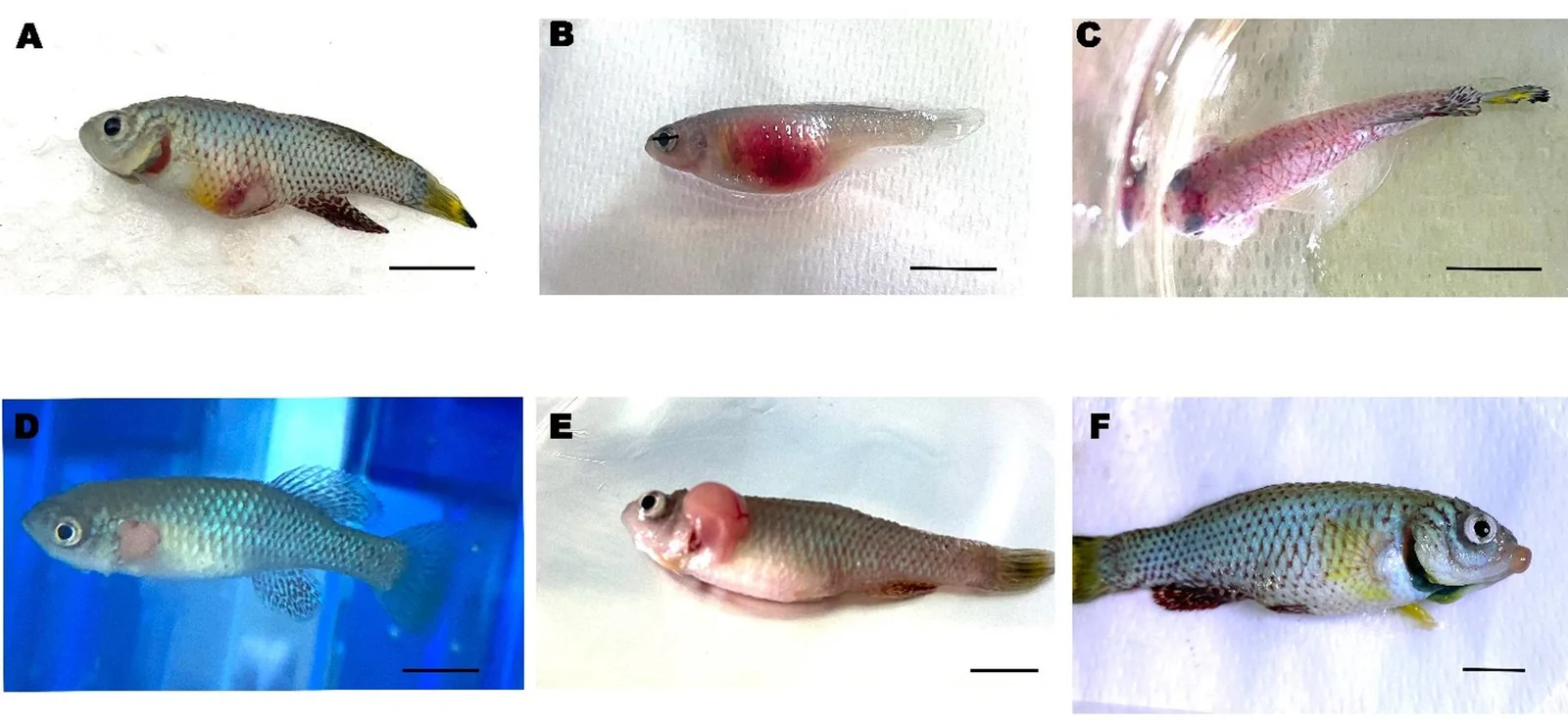
Some frequently occurring clinical signs. (**A**) Adult male with a lesion at the abdominal level. (**B**) Adult female with extensive bleeding between the thoracic and abdominal regions. (**C**) Adult male with cranial petechiae. (**D**) Adult female with an ulcer on the trunk, located immediately behind the operculum. (**E**) Adult male with a neoformation at the opercular level. (**F**) Adult male presenting lip hyperplasia. *Scale bars*: 500 μm.

The explosive growth that characterises this species, peaking between 3 and 15 wph, with mean body mass increasing from ~ 500 mg to ~ 1,000 mg^4,20^, should be explicitly incorporated into welfare assessments of juveniles. By contrast, cutaneous mucus hypersecretion is not reliably assessed at these juvenile stages but may represent a useful clinical indicator of compromised health and welfare in adults^36^. Petechiae and/or haemorrhages (Fig. 7A–C) can occur at all age stages; their occurrence should be interpreted in light of stocking density, environmental enrichment (EE), and overall husbandry conditions. For example, housing two males without physical EE may precipitate intraspecific aggression, leading to integumentary or opercular injuries. Sexually mature females may exhibit abdominal distension, often associated with suboptimal breeding schemes. Other commonly observed clinical signs include skin lesions, such as ulcers or neoformations (Fig. 7D,E), predominantly in old and geriatric fish. Severity depends on the lesion’s location and nature; for instance, masses near the operculum can compromise gas exchange and should be considered a humane endpoint. By contrast, lip hyperplasia (Fig. 7F) occurs frequently in older animals but typically does not impair food intake.

## Discussion

This study reports the results of five years of continuous monitoring in our fish facility aimed at developing a species-specific scoresheet for *N. furzeri*, with particular attention to age-related change in the two most commonly used strains widely used in laboratory, the short- and long-lived strains^12^.

To better assess the welfare-relevant parameters, we have relied on baseline parameters taken from behavioural data already published on these strains^16,20,21,33^. In addition, in our experimental design we have included the calculation of baseline parameters, such as the definition of the opercular beat rate/minutes across age-stages.

Morphometric body-condition indices remain widely used proxies of fish health and colony status. These methods are rapid, inexpensive, and non-invasive, making them well suited to long-term monitoring and repeated measures on the same individuals^31^.

Adequate food intake is essential not only for normal growth and development but also for reproductive success. Observations during feeding provide insight into appetite and motivation to eat. Reduced foraging or food avoidance may signal underlying health problems. Because food consumption naturally declines in old fish, reduced intake is a clear warning sign in juveniles and adults, where it may reflect suboptimal environmental parameters, illness, or social competition^37^.

Respiratory assessment, specifically, the rate, depth, and regularity of opercular movements, provides vital information about physiological status. Deviations may reflect stress, disease, or environmental change; poor water quality, overcrowding, or handling can produce tachypnoea, irregular ventilation, or even apnoea^38^. Opercular beat rate correlates negatively with body weight and increases with water temperature and (to some extent) body size, reflecting metabolic demand; thus, it is influenced by both mass and thermal stress^39^. Temperature, swim-bladder function, and disease also directly affect swimming activity and speed^40^. To our knowledge, quantitative reference ranges for opercular beat rate in *N. furzeri* under standard husbandry conditions are not yet available in the peer-reviewed literature. While opercular movement is widely used in teleost species as a proxy for respiratory activity and physiological stress, species-specific baseline values for *N. furzeri* have not been systematically established. In the present study, we therefore implemented a standardized procedure for the assessment of opercular beat rate, consisting of direct visual counting of opercular movements over a fixed time interval under resting, undisturbed conditions in the home tank. Based on measurements obtained in a representative subset of clinically healthy individuals maintained under standard environmental parameters, we derived preliminary facility-specific reference ranges stratified by age class. These values are intended as an initial operational framework to support practical application of the scoring system.

Monitoring tank occupancy offers a valuable, non-invasive indicator of environmental suitability and behavioural well-being. It is quantifiable and sensitive to environmental change and therefore useful for standardised welfare protocols^34^. Dynamic, varied use of space generally reflects good welfare^35^, particularly at earlier stages. In contrast, stereotyped patterns, such as corner-hugging or avoidance of specific zones, may indicate chronic stress or impaired welfare^41,42^. In aged fish, reduced exploration, increased bottom-dwelling, or social withdrawal, when co-housed, may presage declines in locomotor capacity, sensory function, or social engagement^43,44^. In our observations, we often found healthy fish in the front and center regions of the tank. Although steering clear of certain areas can, in certain situations, relate to stress or less-than-ideal environmental conditions, this action needs careful interpretation. In our experimental setup, tanks were frequently approached from the front during feeding and observation processes, which might have led to a developed spatial preference in otherwise healthy animals. In addition, environmental enrichment (EE) is known to influence spatial distribution and exploratory behaviour in *N. furzeri*^16^. In our facility, fish were maintained under controlled, constant husbandry conditions and the level of structural enrichment was featured by small plastic objects, which may have contributed to a more consistent occupation of specific tank areas. Furthermore, fish exhibiting typical feeding behavior, activity rates, and body condition were consistently noted in these regions, reinforcing the view that this distribution signifies adaptive or context-dependent behaviour rather than compromised welfare. However, we recognize that tank occupancy trends may be affected by various environmental and management factors, and their analysis should consistently incorporate other behavioural and clinical signs. Age-related declines in locomotor activity and functional performance have been described in *N. furzeri*, which exhibits progressive reductions in spontaneous activity and behavioural responsiveness with advancing age^33,45^. Similar, in zebrafish, age-associated hypoactivity and altered spatial preference correlate with markers of neurodegeneration and oxidative stress^46^. Thus, age-stratified monitoring of tank occupancy helps distinguish normal ageing from pathology.

Clinical signs are essential for systematic health and welfare surveillance. In geriatric animals, scoring must be adapted to age-related physiology while distinguishing these changes from pathology, an overlap that makes accurate, context-dependent interpretation crucial^47^.

At a broader level, welfare monitoring and related scoring systems in aged animals should be longitudinal and individualised, as age-related baseline variation must be taken into account. This approach is particularly feasible in *N. furzeri*, where males are typically housed singly, due to their territorial and aggressive behaviour, and females are more commonly maintained in small groups or in breeding setups with one male and multiple females^2,6,48,49^.

In preclinical research, relying on lifespan alone to judge interventions that target ageing can be misleading. Lifespan is shaped by ageing-associated pathologies and by numerous age-sensitive phenotypes, including reduced fertility, neurodegeneration, and sarcopenia and, in some cases, neoplastic lesions reported in *N. furzeri*, although their prevalence remains debated in the literature^10,20,21,50^. Integrating welfare scores into the interpretation of geriatric experiments can therefore enhance translational value, much as routine clinical metrics are integrated in human medicine. Approaches that probe the biology of ageing rather than single-disease models can substantially improve research reliability^51^. As a matter of fact, although not always directly affecting lifespan, age-related changes do require refined, well-suited husbandry and care. Keshavartz and colleagues^51^ frame ageing as “a multidimensional parameter space capturing phenotypic change that takes place between young adulthood and old age”. In this perspective, two aspects of our work are especially relevant: (i) providing a structured and reproducible framework for welfare assessment, based on common baseline parameters and predefined scoring categories. This approach enables consistent evaluation across time points and operators maintaining *N. furzeri*, thereby enabling large-scale data aggregation; (ii) recording changes systematically from early juvenile stage to old age to pinpoint the ages at which key variations emerge, critically given that major transitions can occur early.

Regular, facility-wide evaluation of general health and welfare is fundamental. Routine assessments should not be limited to detecting pain, suffering, or distress related to specific procedures. In *N. furzeri*, this is particularly important given the rapid progression of age-related changes, which requires continuous monitoring of behaviour, appearance, and physiological functions to distinguish normal ageing from impaired welfare.

The use of non-transgenic geriatric animals is increasingly important in ageing studies. While disease-model approaches, particularly in zebrafish, are versatile and highly targeted, they can complicate interpretation when the goal is to understand the biology of ageing per se.

These considerations emphasize the necessity for suitable monitoring approaches in older animals. Improved, welfare-focused monitoring methods have been suggested to detect when age-related alterations might need intervention, relying on agreed-upon and replicable criteria. In this scenario, welfare evaluation must concentrate on the development of pain, suffering, or loss of function, consistent with contemporary ethical and regulatory standards that oversee animal research. On this basis, the proposed scoresheet offers a standardised reporting system that: (i) facilitates colony monitoring by reducing sources of welfare-related and experimental variability in aged fish; (ii) enables multicentre research that includes aged animals; and (iii) eases inter-laboratory transfer during ageing studies. Its utility is strengthened by the choice of *N. furzeri*, now widely recognised as a valuable aquatic model for experimental ageing research. We therefore recommend that facilities breeding and using African turquoise killifish adopt more methodical monitoring and reporting, including detailed husbandry protocols with particular care for old fish.

### Limitations

Our scoresheet derives from direct experience in a single facility; there is no one-size-fits-all solution. Experimental design, strain, workload, and available human resources must be weighed when defining both standard and enhanced monitoring. Accordingly, the scoresheet should be viewed as a starting resource to be refined and integrated into broader frameworks for the optimal management and use of ageing animals.

## Supplementary Information

Below is the link to the electronic supplementary material.

Supplementary Material 1

Supplementary Material 2

Supplementary Material 3

Supplementary Material 4

Supplementary Material 5

Supplementary Material 6

Supplementary Material 7

## Data Availability

The datasets used and/or analyzed during the current study are available from the corresponding author on reasonable request.
