# Supplementary material for: Developing species-specific welfare scoresheets for the African Turquoise Killifish
Source: Sci Rep. 2026 Jun 23;16:28825. doi: 10.1038/s41598-026-58465-3 (PMC13578386; doi:10.1038/s41598-026-58465-3)
Supplement: Supplementary file 1 [file 41598_2026_58465_MOESM1_ESM.docx]

**Age Matters: Developing Species-Specific Welfare Scoresheets for the African Turquoise Killifish**

Elena De Felice^1^*, Antonio Palladino^2^, Aniello Vitale^3^, Ferdinando Flagiello^3^, Maria Raggio^3^, Chiara Attanasio^3^, Paolo de Girolamo^3^, Livia D’Angelo^3^

**Supplementary Video 1: Respiratory pattern.** Adult male resting motionless at the bottom of the tank, showing gasping behaviour characterized by short, labored breaths, often occurring at the surface.

**Supplementary Video 2: Immobility of an old individual.** Old male motionless at the bottom of the tank.

**Supplementary video 3: Swimming activity of a young individual.** Representative swimming activity of a young *Nothobranchius furzeri* individual.

**Supplementary Video 4: Altered swimming activity.** Young/adult exhibiting intermittent loss of balance.

**Supplementary Video 5: Swimming activity of an old individual.** Old male showing progressive decline in activity with age; the fish remains stationary in the middle of the tank.

**Supplementary Video 6: Phenotypic swimming activity of a belly slider.** Young female remaining motionless in a corner for an extended period; when swimming, she stays near the bottom of the tank and exhibits jerky movements.
